# On the Beneficial Effects of the Datura Stramonium, on the Spigelia Marilandica, and on Vermifuge Medicines

**Published:** 1802-11-01

**Authors:** B. S. Barton

**Affiliations:** Philadelphia


### *On the beneficial Effects of the Datura Stramonium, on the Spigelia Marilandica, and on Vermifuge Medicines;* by B. S. Barton, M. D. *of Philadelphia.*

SINCE the publication of the firſt edition of my *Collections*, I have had many opportunities of employing the *Datura Stramonium.* I have uſed it chiefly in the form of an extract, prepared from the freſh leaves. I have principally exhibited it in caſes of mania and epilepſy. I cannot heſitate to ſay that it is a medicine of great and invaluable powers. It is my intention tion to publifh the particulars of the cafes in which I have employed this medicine in a feparate work. I fhall, therefore, content myfelf, in this place, with obferving, that I have found the ftramonium efpecially beneficial in cafes of mania attended with little or no fever, or with a cold fkin and languid circulation. I have thought it neceffary to give the medicine in very large dofes. Beginning with a few grains, the dofe is gradually increafed; and in a few days it may, with fafety, be taken to the extent of fifteen or twenty grains. In one cafe of mania I at length gave it to the extent of fixty grains at a dofe. When the patient had continued upon this dofe for fome time, fhe broke out into biles upon various parts of the body, and was, at length, difcharged from the hofpital perfectly cured. In feveral other cafes of mania the datura has been of effential ufe. Except in one cafe, I have not perceived any inconvenience from it. In this cafe, whilft the patient was taking the medicine to the extent of thirty grains, it produced a very enlarged dilatation of the pupil of the left eye, and a palfy of the palpebra of the fame eye. But even this was only a temporary inconvenience, which was removed in a very fhort time by the application of a blifter The patient refumed the ufe of the extract, and was finally difcharged from the hofpital apparently cured.

The beneficial effects of the ftramonium, in cafes of epilepfy, have been likewife very manifeft. In a cafe of epilepfy, accompanied, at various periods, with fever, the medicine feemed to increafe the fenfe of fulnefs in the head, and other difagreeable fymptoms. But, in feveral other cafes, I exhibited it with the moft manifeft advantage. Although in no cafe have I been able to effect a cure with the ftramonium, I have certainly adminiftered it with the effect of protracting the fits, and of diminifhing their violence. Perhaps much more than this cannot be faid, with a ftrict regard to caution, of any other of the many medicines which have been recommended for the cure of epilepfy.

I have been informed, that in the State of Kentucky the feeds of the ftramonium are fometimes exhibited, with advantage, in cafes of chronic rheumatifm. On this fubject I cannot fay any thing from my own experience. The feeds of this vegetable are unqueftionably endued with very active powers. This is abundantly evident from the pernicious effects which are fo frequently obferved in children who have fwallowed the feeds. Dr. John Archer, of Maryland, has found them of much advantage in cafes of epilepfy. I have ufed them with feeming benefit in a cafe of mania.

*Spigelia*

*Spigelia Marilandica.* In ſome parts of Carolina, &c. this invaluable plant is known, among other appellations, by the name of ſnake-root. It is the *unsteetla* of the Cherokee Indians. Every part of the plant is poſſeſſed of the anthelmintic property, and accordingly, in Carolina, the phyſicians employ the whole plant—chiefly in decoction. But the active power unqueſtionably reſides more eſpecially in the roots. It is the opinion of many perſons, that the deleterious effects which occaſionally occur from uſing this vegetable do not ariſe from any pernicious property inherent in the ſpigelia, but from the root of a diſtinct plant which is often mixed with the ſpigelia. I do not think this notion is entitled to any ſerious attention. The ſpigelia is, without doubt, a poiſonous and narcotic vegetable. It is, in all probability, by virtue of this poiſonous quality, that it proves ſo beneficial in caſes of worms. I am acquainted with a very intelligent phyſician, who, in the exhibition of the ſpigelia, always deems it neceſſary, or proper, to perſevere in the uſe of the medicine until it produces ſome very decided effect upon the brain. I muſt confeſs, however, that I have often found it completely efficacious, without obſerving that it has occaſioned the leaſt inconvenience to the ſyſtem. That it has ſometimes done miſchief, will not, I believe, be denied. Profeſſor Bergius informs us, that he has known inſtances of convulſions cured by the ſpigelia, although no worms were expelled by it. Dr. Garden, ſpeaking of this plant, ſays, 'It eſpecially anſwers in continued or remitting low worm-fevers, in which I uſe its decoction, adding a ſmall proportion of the root of the ſerpentaria virgin. Its effects in abating the feveriſh exacerbations are ſo conſiderable, that in theſe I conſider it as the moſt powerful ſedative. It is an excellent attenuant.' I have been induced to take notice, in this place, of the obſervations of Bergius and Garden, becauſe a pretty extenſive uſe of the ſpigelia has now convinced me that this medicine very often affords relief, and, indeed, effects a cure, in caſes in which worms are ſuppoſed to be preſent, but in which none are diſcharged. If I do not greatly miſtake, this will be found an highly uſeful medicine in ſome of the febrile diſeaſes of children, unaccompanied by worms, eſpecially in the inſidious remittent, which ſo frequently lays the foundation of dropſy of the brain.

*Common Tobacco.* There is a peculiar mode of employing the leaves of the tobacco in caſes of worms, which I cannot avoid mentioning in this place, eſpecially as it has, in many inſtances, produced very happy effects. The leaves are pounded with vinegar, and applied, in the ſhape of a poultice, poultice, to the region of the ſtomach, or other part of the abdomen. In conſequence of this application, worms are often diſcharged, after powerful anthelmintics have been exhibited internally in vain. We ought not to be ſurpriſed at this effect of the tobacco, ſince we know that the ſame vegetable, applied externally, is often very efficacious in inducing vomiting. Accordingly, I have, for ſome years, been in the habit of applying tobacco leaves to the region of the ſtomach of perſons who have ſwallowed large quantities of opium, and other ſimilar articles, with the view to deſtroy themſelves. It is well known, that in theſe caſes the ſtomach is often extremely inirritable, inſomuch that the moſt powerful emetics have little effect in rouſing that organ into action. Here, as an auxiliary at leaſt, the tobacco, uſed in the manner I have mentioned, is certainly very uſeful, and, in many inſtances, ought not to be neglected.

*Melia Azedarach.** When I publiſhed the firſt edition of my *Collections*, I had not *any* experience in the uſe of this vegetable. Since that period, however, I have uſed it in ſeveral caſes of worms, and always with advantage. Indeed, I am inclined to think that the character of this new anthelmintic has not been too highly drawn. I will not aſſert that it ought to be preferred to the ſpigelia; for I have had much more to do with this than with the melia. The melia is unqueſtionably a valuable anthelmintic, and ought to be introduced into general practice. I have employed the bark of the root, both in ſubſtance, and in the ſhape of a ſaturated decoction. In the caſe of an adult, who took the decoction in large quantities, *with the effect of diſcharging great numbers of worms*, it ſeemed to occaſion ſome confuſion of the head, and trembling of the hands. Theſe, perhaps, were accidental ſymptoms; but I am diſpoſed, with the patient, to aſcribe them to the medicine. The worm-caſes in which I have found the melia uſeful, were caſes of the common round worm, or lumbricus inteſtinalis. I have not had any opportunity of trying how far it is a remedy againſt the tænia, or tape-worm. But I am informed that, in Carolina, it has been uſed with the effect of diſcharging great numbers of this ſpecies of worm. Should this prove to be the caſe, the melia will be doubly entitled to our attention as an article of the materia medica. It is not merely in caſes of worms that this vegetable has been found uſeful. Mr. Andrew Michaux, an intrepid

* Pride of India, called in South-Carolina poiſon-berry-tree and China-tree.

intrepid French botanift, informed me, that in Perfia, where this tree grows fpontaneoufly, the pulp which invefts the ftone of the fruit is pounded with tallow, and ufed as an 'antipforic,' in cafes of tinea capitis in children.

Is the melia a narcotic or poifonous vegetable? Its remarkable effects in deftroying and diflodging worms renders this probable, but not certain: for many articles which, with refpect to the human body, are entirely innocent, are known to be noxious to inteftinal worms, and many other animals. Such is fugar, as has been demonftrated by the experiments of Redi, Carminati, and other writers. The cafe which I have alluded to renders the deleterious quality of this vegetable very probable. I may add, that in fome parts of Carolina the root is deemed poifonous. Horfes and horned cattle, however, eat, with impunity, the leaves and berries. Certain fpecies of birds (particularly the turdus migratorius, or robin, and the turdus polyglottos, or mocking-bird) devour the berries in fuch large quantities, that, after eating of them, they are obferved to fall down, and are readily taken. Does not this circumftance render it probable that the berries contain an intoxicating quality? This, however, I believe, is not the general opinion of the inhabitants of Carolina, who afcribe the condition of the birds merely to the circumftance of their having eaten fo abundantly of the berries, that they injure entirely by diftention. The ripe berries have a fweetifh but naufeous tafte.

As the melia is now completely naturalized to the States of Carolina and Georgia, it may not be amifs to clofe this article by obferving, that the fruit of this vegetable is employed in Japan for furnifhing an expreffed oil, which grows hard like tallow, and is ufed for making candles. May not our fellow citizens to the fouth render it worth their attention to follow the example of the Japanefe, in the inftance I have mentioned?

---

It gives us great pleafure to find that the learned author intends to publifh an additional volume relative to other indigenous medicinal productions of the United States, and that he has made fuch progrefs as already to be in poffeffion of fufficient materials.

